# The Association Between Shoulder Pain and Disability Among Saudi Office Workers

**DOI:** 10.7759/cureus.48052

**Published:** 2023-10-31

**Authors:** Abdullah H Alzahrani, Bijad Alqahtani

**Affiliations:** 1 Department of Rehabilitation Sciences, College of Applied Sciences, Shaqra University, Shaqra, SAU

**Keywords:** saudi arabia, office workers, screen time, shoulder disability, shoulder pain

## Abstract

Background and objective

Shoulder pain stands out as the most prevalent musculoskeletal issue that office workers face. This type of pain has been observed to be linked to various aspects of one's job. To address this concern, the current research endeavors to examine the impact of digital device use on the intensity of shoulder pain and the extent of disability experienced by office employees in Saudi Arabia. This study is driven by two principal objectives. Firstly, it aims to assess the overall occurrence of shoulder discomfort and functional limitations among the Saudi office worker population. Secondly, it seeks to draw comparisons between the parameters of the shoulder pain and disability scale and the amount of time spent using electronic screens.

Methodology

This cross-sectional descriptive study was conducted in Riyadh, Saudi Arabia. We recruited 150 participants to measure shoulder pain and disability among office workers. The study was carried out to determine the general frequency of shoulder discomfort and impairment by using the Shoulder Pain and Disability Index (SPADI). The survey was conducted online in June 2022 via Google Forms. The survey questions included data regarding age, sex, year of study, exercise, and duration of computer time per day (hours).

Results

This study recruited 150 participants to measure shoulder pain and disability among office workers. The mean age of the cohort was 42.56 ± 2.56 years. Among the total participants, 90 (60%) were male, and 60 (40%) were female. We observed that pain parameters had a negative correlation of -0.008 with screen time. The participants who had high shoulder pain and disability scores were older in age and spent less than two hours on screens, which affects the correlation results, and hence we recommend performing a study involving the younger population working on screen for more than four hours to find the better correlation. At the same time, the shoulder disability score reported a correlation of 0.05, and the overall correlation between SPADI and screen time was observed to be 0.04. Based on these findings, the present study observed a weak correlation between SPADI and screen time.

Conclusion

These results suggest that while there may be some association between screen time and shoulder pain and disability, it is not substantial. Therefore, it is unlikely that screen time alone significantly contributes to the occurrence or severity of shoulder pain and disability among office workers. Additional factors and variables may need to be explored in future research to gain a more comprehensive understanding of this issue.

## Introduction

Musculoskeletal pain is the primary cause of disability in developed countries and is a serious public health issue with considerable economic ramifications [1]. The prevalence of work-related musculoskeletal illnesses has increased globally, partly due to the expansion of office-based employment and the use of digital devices [2]. The frequency of neck and upper-limb discomfort among office workers is highest in European countries, where over 30% of workers report having low back pain [3-5].

Office workers spend more than four hours per day or 20 hours per week on devices that involve data display at work [6]. They are considered a population at risk of long-term musculoskeletal problems that cause pain and disability [3,7]. It has been demonstrated that occupational characteristics such as maintaining static positions for long periods or using improper postural and ergonomic hygiene, as well as the work environment and bad workstation design, are linked to work-related musculoskeletal problems among office employees [7-9]. Joint inflammation caused by work is indicated by muscular pains, weaknesses, job-related pain, arthritis, the need to adjust one's working posture, and persistent muscular stiffness [10]. Musculoskeletal diseases can be caused by prolonged sitting, computer work, repetitive motions, static postures, and poor environmental circumstances, to name a few [11]. Musculoskeletal disorders ranked second among health issues in 1997 according to the National Institute for Occupational Safety and Health (NIOSH) [12]. In the US, musculoskeletal diseases cause about one million people to miss work each year to receive treatment and relief from pain, and the government compensates 2% of the workforce for backaches each year [12]. The prevention and control of occupational illnesses receive considerable attention today due to the prevalence of musculoskeletal ailments, which results in significant compensation being paid to the workforce [13,14].

Shoulder pain is a common musculoskeletal disease. In affluent countries, 67% of people will experience shoulder pain at some point in their lives [15]. A significant portion of physiotherapy appointments pertain to addressing this specific medical condition [16]. This condition is believed to have one of the longest median recovery periods among all musculoskeletal injuries, leading to substantial personal and financial losses resulting from reduced functional capacity and employment opportunities [17]. Digital gadget use undoubtedly has numerous benefits, but if improperly used, it can also result in significant health issues, such as musculoskeletal and ophthalmic problems [18]. Many people have reported experiencing musculoskeletal pain when using a laptop, with neck and shoulder pain being the most prevalent [19]. Other problems include tingling in the fingers and backaches.

Shoulder pain is a prevalent musculoskeletal issue that frequently afflicts office workers, with potential implications for their job performance and overall well-being. Investigating these aspects in a specific occupational context can contribute significantly to the existing body of knowledge on musculoskeletal issues among office workers. Properly understanding the impact of screen time on shoulder pain and disability can guide employers in adopting effective workplace strategies. Employers can focus on broader ergonomics, by promoting frequent breaks and encouraging proper posture rather than solely addressing screen time as a primary concern. Manufacturers and designers can use these findings to create more ergonomic office equipment and furniture. This may include chairs, desks, and computer peripherals that are better suited to reducing the risk of shoulder pain and discomfort. Equipping individuals with knowledge about self-care practices can contribute to minimizing shoulder pain and disability. This study aimed to determine how the use of digital devices influences the severity of shoulder pain and disability among Saudi office employees. The study focuses on two primary objectives: to evaluate the general frequency of shoulder discomfort and impairment among Saudi office workers and to correlate the parameters of shoulder pain and disability scale with the amount of screen time.

## Materials and methods

This descriptive cross-sectional study was conducted among office workers in the Riyadh Region. The survey was conducted online in June 2022 via Google Forms. The participants were selected by convenient sampling and provided their written informed consent through an online survey. The data were collected anonymously. Potential respondents were provided with all relevant information about the ethical and moral aspects of the study at the outset. The study was conducted after obtaining approval from the ethics committee. The inclusion criteria were as follows: patients of both genders aged 18 years and older, who were employed full-time in office settings, irrespective of the type or severity of their shoulder pain. All cases that had undergone recent neck surgery or experienced trauma and vertebral abnormalities were excluded from the research, as these factors could make it challenging to attribute observed neck pain solely to digital device usage or workplace factors. The survey questions included information about age, sex, year of study, exercise, and duration of computer time per day (hours).

Study tool

The Shoulder Pain and Disability Index (SPADI) [20] was used in this research. The SPADI has two subscales: a "pain" subscale and a "disability" subscale. The subscales include five items for "pain" and eight items for "disability," which pertain to different shoulder issues participants have experienced during their jobs. Participants can rate each item on the visual analog scale used in this study on a scale ranging from "no pain", and "no difficulty" to "worst pain imaginable", and "so difficult required help." The average of each section's item scores is calculated to obtain independent subscale scores ranging from 0 to 100. The two subscale values are then averaged to generate a SPADI total score, which ranges from 0 (best) to 100 (worst). No SPADI score is computed if more than two items on a subscale go unanswered.

To avoid the copyright issue, we only used SPADI variables, and not the complete sentences. Items were translated into Arabic, and two translation teams with Arabic as their mother tongue completed the first forward translations. Then, two back-translations by native English speakers employed as professional translators were generated. Two bilingual translators assisted in the translating process, and the questionnaire was tested first at the administration offices of our own university (10-15 participants voluntarily participated). A committee reviewed the source and final versions. No serious translation issues were discovered in the SPADI's forward and back translations, and a preliminary Arabic version was agreed upon. This version was pre-tested among a sample of patients before a final version was made accessible for the current investigation. The sample size was measured for the organization that the study recruited from. They had more than 3000 workers working morning and evening shifts, and, for the purpose of feasibility, we only chose the morning shift office workers, and that is why the correlation we found was observed. They had proper sleep. However, we also recommend performing a study on evening workers to establish a better correlation.

The translations aimed to maintain the original meaning of the SPADI items, which required careful consideration of language nuances and context. The translation process took into account cultural differences to ensure that the translated items were culturally appropriate for the target population. Efforts were made to ensure that the translated items were clear and comprehensible to Arabic-speaking participants to avoid any potential ambiguity. The pre-testing phase was deemed essential to identify any potential difficulties or misunderstandings that participants might encounter with the translated items, so that necessary revisions could be made.

Statistics analysis

The collected data were evaluated using IBM SPSS Statistics (IBM Corp., Armonk, NY). The mean of screen time, the SPADI pain scale, the SPADI disability scale, and the overall SPADI score were calculated as they were all continuous variables. In stable patients, the within-patient standard deviation (SD), obtained from a one-way analysis of variance (ANOVA), was used to calculate SPADI reproducibility. The correlation between screen time and SPADI scores was examined using Pearson correlation.

## Results

This study involved a total of 150 office workers. We wanted to look into their shoulder pain and how it affected their ability to work. The mean age of the participants was 42.56 ± 2.56 years. Out of the 150 participants, 90 were men, which is 60% of the total, and 60 were women, making up 40% of the total participants. As for occupation, there were 30 teachers, accounting for 20% of the total, while accountants made up 10% with 15 participants. Data entry operators constituted the most common type of workers, with 55 participants, accounting for 36.6% of the total. Those working in the banking sector were 25 in number, comprising 16.6% of the total participants. Lastly, there were 15 businessmen, making up 10%. Additionally, there were 10 participants in other job roles, making up 6.6% of the total (Table 1).

**Table 1:** Demographic characteristics of study participants SD: standard deviation

Variable	Total number (N)	Percentage (%)
Age, years, mean ± SD	42.56 ± 2.56	
Sex		
Male	90	60%
Female	60	40%
Occupation		
Teacher	30	20%
Accountant	15	10%
Data entry operator	55	36.6%
Banking	25	16.6%
Business	15	10%
Others	10	6.6%

The working hours in Saudi Arabia are from 8 a.m. to 3 p.m., with a lunch break from 12 p.m. to 1 p.m. Therefore, the present study divided screen time into the following three categories: less than two hours, less than four hours, and less than six hours. The overall shoulder pain as per the SPADI score was 26.4 ± 5.7 among participants with less than two hours of screen time. Comparatively, the mean score among participants with screen time of more than two hours was high (24.7 ± 5.9 and 25.7 ± 7.4 for screen time ≤4 hours and screen time ≤6 hours, respectively); however, this difference was not statistically significant (F=0.965; p>0.05). The shoulder disability was also measured based on the same criteria. The present study observed that participants with a screen time of ≤6 hours had a SPADI score of 37.5 ± 8, which was higher compared to those with a screen time of ≤2 hours (36.8 ± 8), as well as those with screen time of ≤4 hours (35.8 ± 7.5). However, no statistically significant difference was found between the groups in this regard (F=0.59; p=0.55) (Table 2).

**Table 2:** Comparison of SPADI scores with respect to screen time SPADI: Shoulder Pain and Disability Index; SD: standard deviation

Variables	Screen time ≤2 hours, mean ± SD	Screen time ≤4 hours, mean ± SD	Screen time ≤6 hours, mean ± SD	ANOVA (F)	P-value
SPADI pain score	26.4 ± 5.7	24.7 ± 5.9	25.7 ± 7.4	0.965	0.38
SPADI disability score	36.8 ± 8	35.8 ± 7.5	37.5 ± 8	0.59	0.55
Overall SPADI score	63.2 ± 8.9	60.5 ± 7.7	63.2 ± 9.5	1.66	0.19

Comparing the parameters of pain in the SPADI scale, the present study found that participants with a screen time of less than two hours had more pain than other groups (5.08 ± 2.31 vs. 3.88 ± 1.91 and 3.5 ± 1.9). The higher pain score could be attributed to the higher number of older participants in the group with less than two hours of screen time. The difference between the groups in this regard was statistically significant (F=7.95; p=0.0005). However, no other parameters showed statistical significance. Furthermore, the participants with ≤6 hours of screen time reported pain while touching the back (6 ± 2.24), reaching for something on a high shelf (5.9 ± 2.2), and pushing with the involved arm (5.8 ± 2.4). There was a statistically significant difference between the groups with regard to pushing with the involved arm (F=3.38; p=0.03) (Table 3).

**Table 3:** Comparison of SPADI pain parameters with respect to screen time SPADI: Shoulder Pain and Disability Index; SD: standard deviation

Parameters	Screen time ≤2 hours, mean ± SD	Screen time ≤4 hours, mean ± SD	Screen time ≤6 hours, mean ± SD	ANOVA (F)	P-value
Pain at its worst?	5.08 ± 2.31	3.88 ± 1.91	3.5 ± 1.9	7.95	0.0005
Feeling pain when lying on the affected side?	5.24 ± 2.25	4.74 ± 2.3	4.38 ± 2.46	1.65	0.19
Feeling pain when trying to reach something up high on the shelf?	5.6 ± 2.2	5.3 ± 2.4	5.9 ± 2.2	0.93	0.39
Feeling pain putting my hand on the neck's back?	5.6 ± 1.8	5.7 ± 2	6 ± 2.24	0.45	0.63
Feeling pain when using the affected arm to push?	4.7 ± 2.1	4.9 ± 1.9	5.8 ± 2.4	3.38	0.03

Comparing the shoulder disability parameters of the SPADI scale, the study revealed that participants with a screen time of ≤6 hours had more difficulty in washing hair than the other groups (5.1 ± 2.1 vs. 3.6 ± 1.9 and 4.7 ± 2.3), and the difference was statistically significant (F=6.47; p=0.002). Furthermore, a statistically significant difference was observed between the three groups with regard to wearing button-down shirts (F=4.69; p=0.01) (Table 4).

**Table 4:** Comparison of shoulder disability parameters among groups SD: standard deviation

Parameters	Screen time ≤2 hours, mean ± SD	Screen time ≤4 hours, mean ± SD	Screen time ≤6 hours, mean ± SD	ANOVA (F)	P-value
Washing your hair?	3.6 ± 1.9	4.7 ± 2.3	5.1 ± 2.1	6.47	0.002
Washing your back?	5.1 ± 1.9	5.4 ± 1.9	5.12 ± 2.1	0.3	0.74
Putting on an undershirt or jumper?	4.5 ± 1.8	4.9 ± 2.04	4.9 ± 1.8	0.65	0.52
Putting on a shirt with front buttons?	4.2 ± 2.1	3.1 ± 1.5	3.7 ± 1.8	4.69	0.01
Putting on your pants?	4.1 ± 2	3.3 ± 1.8	3.9 ± 1.8	2.18	0.11
Putting something up on a high shelf?	4.9 ± 1.9	4.7 ± 2.1	4.6 ± 2.0	0.21	0.8
Disability when carrying a 4.5-kilogram (10-pound) heavy object	5.1 ± 2.1	4.8 ± 2.1	5.1 ± 2.1	0.36	0.69
Disability while taking a small object out of your back pocket	5 ± 1.8	4.7 ± 2.1	4.9 ± 2	0.14	0.86

The present study observed that pain parameters had a negative correlation of -0.008 with screen time. However, the shoulder disability score had a correlation of 0.05, and the overall correlation of SPADI with screen time was observed to be 0.04. Based on these findings, the present study observed a weak correlation between SPADI and screen time.

## Discussion

The study aimed to determine how frequently office workers use screens and whether this use is linked to shoulder discomfort and disability. When evaluating pain and disability in people with shoulder pain, the SPADI proved to be a reliable tool. The participants were given a soft copy of a self-structured questionnaire, which they were requested to complete by documenting their level of pain and handicap. A study by Côté et al. [21] looked into workers' most significant risk factors for neck-shoulder pain (NSP). They reported that the high demands for forceful labor are the major risk factors. The present study observed that participants with screen time ≤6 hours had a SPADI disability score of 37.5 ± 8, which was higher than the other groups. Furthermore, when comparing the disability scores of the other two groups (screen time ≤2 and ≤4 hours), the score was higher in participants with screen time of less than two hours (36.8 ± 8 vs. 35.8 ± 7.5). However, this difference was not statistically significant (F=0.59; p=0.55). These findings align with the study conducted by Cho et al. [22], which observed a similar trend among individuals who used screens excessively, experiencing shoulder pain and impairment. Among computer users, the most prevalent regions of musculoskeletal symptoms were the shoulder (73%), neck (71%), and upper back (60%) areas.

In the present study, the shoulder disability score showed a correlation of 0.05, and the overall correlation of SPADI with screen time was observed to be 0.04. Hence, the present study observed a weak correlation between SPADI and screen time. These results are in line with the study by Silva et al. [23], whose study involved a total of 969 students aged between 13 and 19 years. These participants were asked to fill out a questionnaire regarding chronic pain, their engagement in moderate and vigorous physical activity, the amount of time spent on screen-based activities such as watching television (TV) or digital versatile discs (DVD), playing, using mobile phones and computers, as well as their sleeping hours. They discovered a correlation between screen use and pain in the shoulder, neck, upper back, and lower back (p=0.04). Moreover, the current study's findings are supported by a study by Costigan et al. [24], which reported a connection between young female office workers' screen usage and musculoskeletal pain. The results of our investigation also suggested a shaky correlation between screen time and shoulder dysfunction.

The results of a cross-sectional survey conducted in 2016 among university students, staff, and faculty lend validity to our findings. The prevalence of symptoms while using a device was found to have increased, mainly in the neck and upper back region [25]. A study by Hallman et al. [26] investigated the relationship between blue-collar employees' subjectively reported sitting duration and neck and shoulder pain. According to their findings, blue-collar employees who spend a lot of time sitting have a higher risk of developing neck and shoulder pain [26]. Another study, by Torsheim et al. [27], found that screen-based activities are a predictable contributing factor to real health complaints, particularly musculoskeletal disorders (p=0.01). A cross-sectional analysis of 120 participants (all of them financiers) showed that 48.33% of respondents had shoulder pain, and 71.67% had neck pain. Only 3% of their seats had immovable armrests, while 25% of brokers used movable armrest seats [28]. The current study's findings also align with those of a survey among young people in Finland, which shows a connection between neck and shoulder pain and TV and PC use. The time spent on screen-based activities was explicitly mentioned as a risk factor, and a weak attachment suggests that these activities promote real complaints. Therefore, it has been assumed that altering improper working habits and behavior will reduce significant risk. Additionally, ergonomically changing the workplace will significantly contribute to preventing these health issues [29,30].

In the current study, age and years of experience were important factors. Office workers from Saudi Arabia and other countries have demonstrated similar results [31,32]. Older individuals may be more susceptible to shoulder discomfort for several reasons. Firstly, aging often brings about degenerative changes in musculoskeletal structures, including the shoulder joint. These changes can result in increased vulnerability to pain and discomfort. Additionally, as people age, their muscle mass and strength may decrease, making them more prone to musculoskeletal issues. Years spent in an office job can contribute to the observed relationship between SPADI and screen time. Prolonged exposure to poor ergonomics, such as improper workstation setup, inadequate chair support, and repetitive tasks, can lead to cumulative wear and tear on the shoulder muscles and joints. Over time, this can result in chronic discomfort and impairment. While the study shows a correlation between screen time and SPADI, not all individuals will experience the same level of discomfort or disability. Factors such as posture, ergonomic adjustments, and pre-existing health conditions can vary among participants and influence their response to screen time.

In Saudi Arabia, prevalence rates of shoulder pain and disability for dentists and radiologists were reported to be 77.9 and 88.9%, respectively [33,34]. In contrast, the rates for office workers from three big corporations ranged from 39% to 51% [32]. The rates of those seeking medical counsel are far greater globally [35] than those currently represented in our sample, even though no local studies have engaged in proper follow-ups [32-34]. These sample populations may be more aware of the potential advantages of treatment because they included people with medical training and those who worked in the healthcare industry. Additionally, the evaluated office workers' unwillingness to seek medical attention could have been caused unintentionally by social circumstances. Research has indicated that Saudis rarely seek medical attention unless their illness has progressed to an advanced stage or their symptoms have worsened [36,37]. This argument holds true despite the size and density of healthcare facilities and the nation's free healthcare system. Between 65 and 80% of Saudis claim to use complementary and alternative therapies for musculoskeletal disorders [38].

This study has a few limitations, such as its relatively small sample size of 150 participants, which may limit the generalizability of its findings to a broader population. The data collected in our study relied solely on self-reported information, which could be subject to recall bias or inaccuracies. The cross-sectional nature of the study limited our ability to establish causal relationships between screen time and shoulder pain and disability. The study was conducted in Riyadh, Saudi Arabia, and may not represent the experiences of office workers in other regions. Further research with larger and more diverse samples and longitudinal designs may provide a deeper understanding of the relationship between screen time and shoulder health among office employees.

## Conclusions

Our study demonstrated that office employees who spend more than two hours in front of a screen typically experience shoulder discomfort symptoms, and those who spend ≤6 hours frequently experience shoulder disability. There was a higher incidence of pain and impairment symptoms among older workers, those with more years of experience, and those who were overweight. The findings of this study could be used to devise strategies and interventions that would reduce the prevalence of shoulder pain and other indicators of impairment among office employees.
